# Supplementary material for: Renal Ischemia Tolerance Mediated by eIF5A Hypusination Inhibition Is Regulated by a Specific Modulation of the Endoplasmic Reticulum Stress
Source: Cells. 2023 Jan 25;12(3):409. doi: 10.3390/cells12030409 (PMC9913814; doi:10.3390/cells12030409)
Supplement: Supplementary file 1 [file cells-12-00409-s001.zip › cells-2113434-supplementary.pdf]

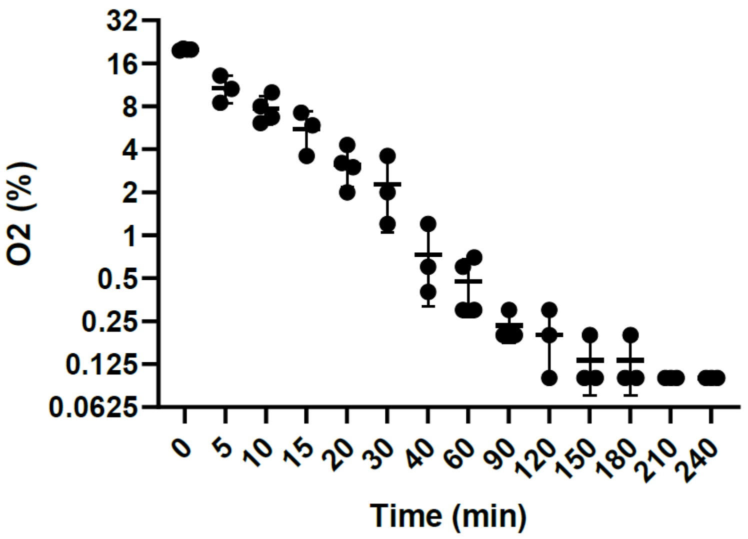

Supplementary figure S1. Time course of O_2 level decrease after switching to a 100% N_2 atmosphere.

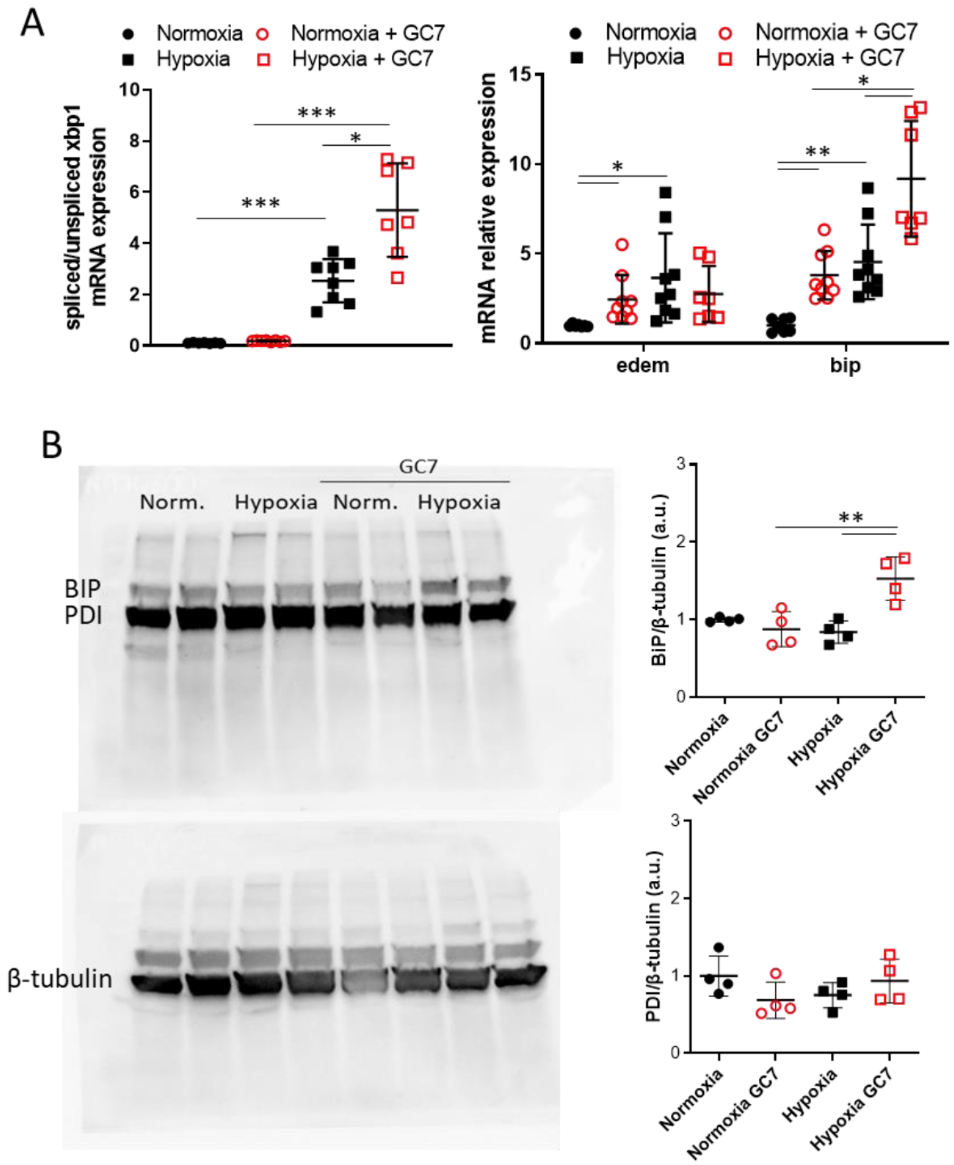

Supplementary figure S2: GC7 increases XBP1 splicing and BIP expression under mild hypoxia. PCT cells pretreated for 8 hours either with a vehicle or with 30 μM GC7 and 24 hours later, exposed to normoxia or mild hypoxia (1% O_2) for 24h. At the end of the experiment, mRNA (A) and protein (B) expressions were measured. A) Scatter plot representation of individual values along with mean ± SD for *bip*, *edem* and the ratio spliced/unspliced *xbp1* mRNA. B) Representative western blots of BiP and PDI protein levels along with the scatter plot representation of individual values with mean ± SD for respective blots. *= p≤ 0.01; **= p≤ 0.001; ***= p≤ 0.0001.

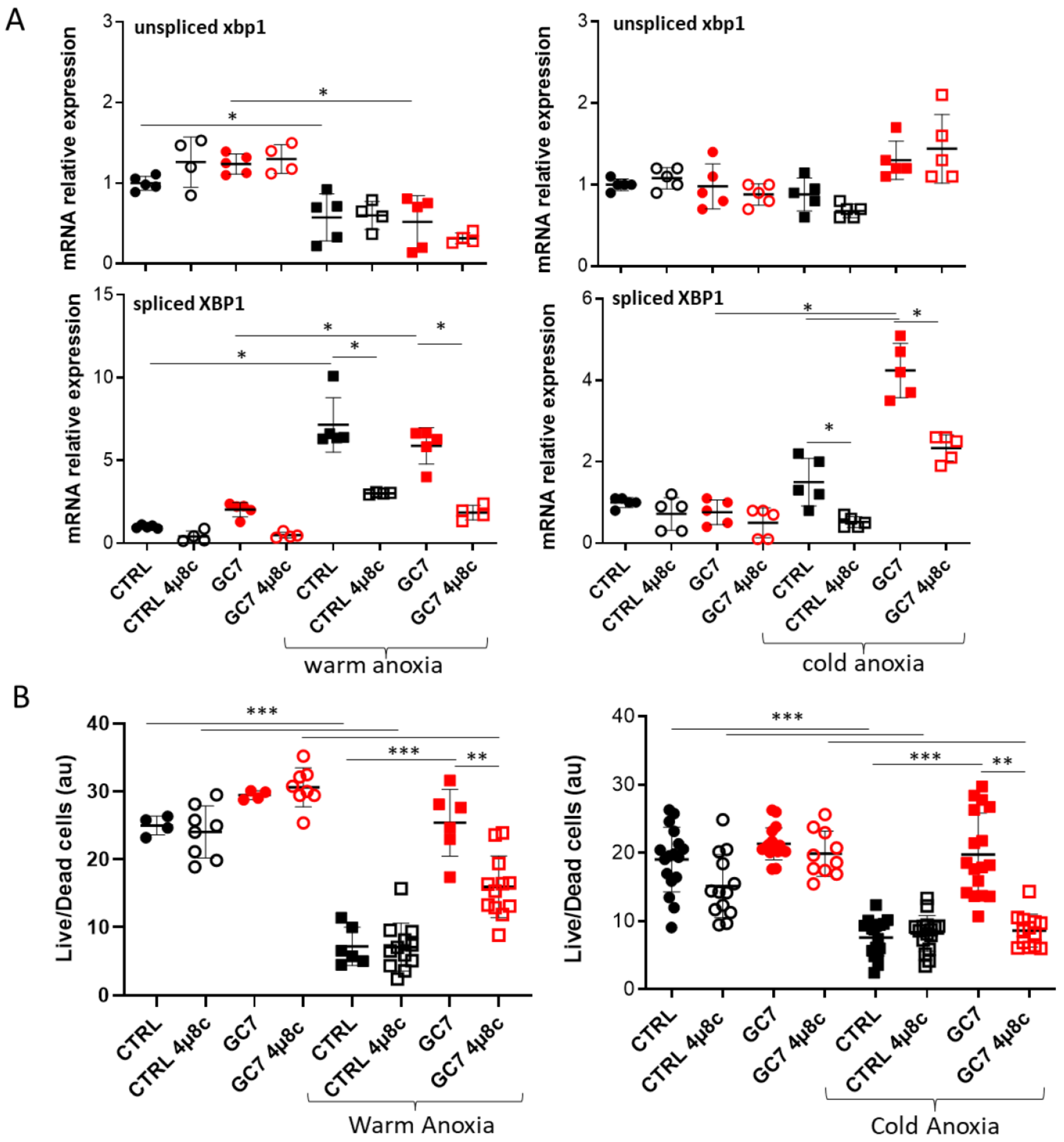

Supplementary figure S3: Impact of 4μ8c treatment on survival and splicing of XBP1. PCT cells pretreated for 8 hours either with a vehicle or with 30 μM GC7 and 24 hours later, exposed to either warm anoxia (≤0.1% O_2 at 37°C for 24h) or cold anoxia (≤0.1% O_2, 4°C, for 16 h), in presence or not of 1μM IRE-1*α* inhibitors, 4μ8c. At the end of the experiment, (A) mRNA expressions of spliced and unspliced *xbp1* were determined and used to calculate the ratio displayed in Figure 6, and (B) cell viability was evaluated using a live/dead fluorescence assay to evaluate survival rate displayed in figure 5. Scatter plot representation of individual values along with mean ± SD. * = p≤ 0.01; **= p≤ 0.001; ***= p≤ 0.0001.
